# Survivors’ experiences of informal social support in coping and recovering after the 2017 Manchester Arena bombing

**DOI:** 10.1192/bjo.2022.528

**Published:** 2022-07-04

**Authors:** John Drury, John Stancombe, Richard Williams, Hannah Collins, Lizzie Lagan, Alan Barrett, Paul French, Prathiba Chitsabesan

**Affiliations:** School of Psychology, University of Sussex, UK; Young People's Mental Health Research Unit, Pennine Care NHS Foundation Trust, UK; Welsh Institute for Health and Social Care, University of South Wales, UK; Complex Trauma and Resilience Research Unit, Greater Manchester Mental Health NHS Trust, UK; Oldham Healthy Young Minds, UK; Manchester Resilience Hub, Pennine Care NHS Foundation Trust, UK; and School of Health Sciences, University of Salford, UK; Research and Innovation Department, Pennine Care NHS Foundation Trust, UK; and Faculty of Health, Psychology and Social Care, Manchester Metropolitan University, UK; Young People's Mental Health Research Unit, Pennine Care NHS Foundation Trust, UK; and Faculty of Health, Psychology and Social Care, Manchester Metropolitan University, UK

**Keywords:** Psychosocial care, support group, terrorism, Manchester Arena attack, social support

## Abstract

**Background:**

Much of the psychosocial care people receive after major incidents and disasters is informal and is provided by families, friends, peer groups and wider social networks. Terrorist attacks have increased in recent years. Therefore, there is a need to better understand and facilitate the informal social support given to survivors.

**Aims:**

We addressed three questions. First, what is the nature of any informal support-seeking and provision for people who experienced the 2017 Manchester Arena terrorist attack? Second, who provided support, and what makes it helpful? Third, to what extent do support groups based on shared experience of the attack operate as springboards to recovery?

**Method:**

Semi-structured interviews were carried out with a purposive sample of 18 physically non-injured survivors of the Manchester Arena bombing, registered at the NHS Manchester Resilience Hub. Interview transcripts were thematically analysed.

**Results:**

Participants often felt constrained from sharing their feelings with friends and families, who were perceived as unable to understand their experiences. They described a variety of forms of helpful informal social support, including social validation, which was a feature of support provided by others based on shared experience. For many participants, accessing groups based on shared experience was an important factor in their coping and recovery, and was a springboard to personal growth.

**Conclusions:**

We recommend that people who respond to survivors’ psychosocial and mental healthcare needs after emergencies and major incidents should facilitate interventions for survivors and their social networks that maximise the benefits of shared experience and social validation.

In May 2017, a suicide bomber detonated an improvised explosive device in the foyer of the Manchester Arena as people were leaving a concert by the pop singer Ariana Grande. Around 19 500 people were present at the Arena, and the blast killed 23 people including the bomber and injured over 230 others.^1^ Many of the people killed and injured were children or their family members.^2^ Much of the psychosocial care received by people affected by disasters and major incidents such as the Manchester Arena attack is informal, and is provided by families, friends, peer groups and social networks.^3^ There is a need to understand and facilitate this informal social support, for three key reasons: effective social support is crucial for coping and recovery for most survivors,^4^ formal services do not meet all survivors’ needs^4,5^ and effective psychosocial care has been shown to prevent people developing mental health disorders.^6^ The present paper contributes to this understanding through an interview study with survivors of the Manchester attack. We sought to answer three questions. First, what is the nature of any informal support-seeking and provision for some of those who experienced the Manchester Arena terrorist attack? Second, who provided support, and what makes it helpful? Third, to what extent do support groups based on shared experience of the attack operate as springboards to recovery?

## Needs for psychosocial and mental healthcare following disasters and major incidents

Terrorist-related mass casualty incidents have increased in recent years in the UK and other countries.^7^ These incidents lead to a range of mental health outcomes for people affected. A minority of people affected develop substance misuse, anxiety disorders, depression and post-traumatic stress disorder (PTSD).^8–11^ However, most survivors require psychosocial care to deal with their distress, not specialist mental health treatment.^11,12^ In a recent paper, we emphasised the importance of psychosocial interventions because a substantial number of affected people continued to struggle or to be distressed several years later, despite not reaching diagnostic thresholds.^13^ In addition, people who suffer from PTSD are likely to need psychosocial care in parallel with specialist mental health treatments. The need for non-specialist psychosocial care that is informed by the principles of psychological first aid^14^ in relation to disasters is now widely accepted, and is recognised in formal models such as the North Atlantic Treaty Organization's stepped model of care,^15^ and the model advocated in 2021 by NHS England.^16^

## The nature and consequences of social support

We are clear about the huge importance and power of social support as a key component of psychosocial care to promote how people deal with and recover from major incidents.^15,17–19^ Social support can take different forms, including practical (or ‘tangible’ or ‘instrumental’), emotional and informational support.^19–21^ Emotional support has been defined as actions that show concern and make survivors feel cared for or loved.^22^ In everyday life, social support helps people to cope with work stressors,^23^ and can protect people's health.^24^ During an emergency, emotional support from responders can enable people to endure difficult public health interventions.^25^ In the recovery period after disasters, social support contributes toward survivors’ well-being,^4,17^ and helps to reduce psychiatric symptoms.^26,27^

## Sources of social support

Much of the care that people receive after terrorist attacks and other emergencies takes place informally, in the form of social support from their families, friends, peer groups and social networks.^4,17,18^ Indeed, after the London bombings of 7 July 2005, many Londoners said they preferred this approach to professional care.^28^ In addition, many people affected by incidents such as the Manchester Arena bombing do not come forward for professional support, or they come forward months later because their distress persists or they develop symptoms of psychiatric disorders.^1^ In the meantime, they are dependent on informal social support, whatever their preferences.

Consequently, we differentiate formal and informal support. By the former, we denote support designed and delivered by agencies that are commissioned to deliver forms of care for survivors of incidents. By contrast, the latter refers to social support that is arranged by survivors for themselves or in combination with other people who may be similarly affected. It includes spontaneous support from friends and families. Support provided by peers is one form of social support. It may be formal or informal. Basset et al make a similar distinction between informal, formal and peer-led support.^29^

## Mechanisms of social support

The question of how social support works to produce positive effects appears to be linked to that of who gives that support. On one level, sources can be very different: some are face to face, whereas others may be online; some are with people to whom survivors are already close, whereas others may be with strangers; and some encounters may be structured, whereas others are spontaneous. But on another level, these different forms of informal support may share in common the fact that they are not organised or delivered by professional or expert sources. Moreover, some of these forms of informal support may have one further key feature in common that unites them in the minds of survivors and differentiates them from professional sources of support: shared experiences of the disaster.

A number of different lines of research each suggest that survivors particularly value support from those people who have had similar experiences of the ‘trauma’ to themselves. For example, after the 2011 terrorist attack in Utøya, Norway, bereaved parents particularly valued support from other parents bereaved in the incident.^30^ Similarly, a study of people affected by the mass shooting at Columbine High School in 1999 found that support from people who had a similar experience to themselves was more effective than support from the ‘outside’.^31^ These ‘similar’ others provided validation and a space to share emotions. Other recent studies of informal support for survivors of traumatic events found that peers who shared similar experiences were able to provide sense-making and a feeling of unity,^32^ whereas attempts at support from the ‘outside’ sometimes displayed a lack of understanding.^33^

## Peer support

In some cases, survivors form groups with peers who share their experiences of the disaster or major incident.^3,32^ Although there are different definitions of peer support, they all emphasise support from others who are like oneself, and in understanding participants as active rather than ‘passive victims’.^34,35^ Peer support may be organised formally or arise informally. Watkins identifies three ways that peer support groups can be initiated and facilitated: vertical groups that ‘are initiated and facilitated by professional service providers’, horizontal groups that are ‘initiated by and for those directly affected’ and multidimensional groups that are ‘initiated and facilitated by those with previous personal experience of disaster for those with newer experience’.^35^ During the COVID-19 pandemic, for example, one author (R.W.) developed peer support groups for National Health Service (NHS) staff that are mainly vertical.^36^

## Support through psychological membership of groups

The ‘social cure’ approach^37,38^ agrees with the peer group support literature on the benefits of psychological group membership, but suggests some specific mechanisms for these benefits. Specifically, the social cure approach suggests that identification with (and not just membership of) the group increases expectations of social support, motivations to provide support to fellow group members and optimal interpretations of offers of support.^38^ In addition, the approach suggests that shared social identity can provide validation for beliefs, emotions and identity itself.^39^ These benefits apply not only to face-to-face groups, but also to dispersed social networks (e.g. online groups); from this perspective all are ‘groups’, since the psychological basis in each case is self-categorisation as a group member.^37^

The links between shared social identity, support and recovery following a disaster are illustrated in the studies by Ntontis and colleagues of people affected by the floods in York, UK in 2015. Interviews with 17 affected residents found that emergent shared community identity was a basis of social support given to and expected from others locally.^40^ A survey of 431 residents found that shared social identity based on the common experience of the flood was associated with well-being via expected support.^41^ Similarly, in a study of experiences in the aftermath of the 2011 Great East Japan Earthquake, identification with a group was found to be associated with perceptions of greater emotional support, which, in turn, enhanced post-disaster community cohesion, an indicator of well-being.^42^

Other research on the role of shared social identity in disasters has found that positive effects can go beyond well-being. Muldoon et al's study of earthquake survivors in Nepal found that shared experience of the earthquake enhanced collective efficacy, which, in turn, predicted post-traumatic growth.^43^ Here, being psychologically part of the ‘traumatised’ group was a springboard to development beyond the group.

However, not all groups are good for mental health, and some social identities can be a ‘curse’ rather than a cure.^44^ Thus, some argue that people need eventually to ‘move on’ from the disaster, and that a decline in ‘victim’ or ‘survivor’ identity is therefore part of the recovery process.^45^ There is a risk that if someone feels that no outsiders can understand their suffering, the person's ability to form new supportive relationships beyond the group could become closed off. In this argument, by sustaining an unhealthy focus on the trauma, such a group is not a springboard but an anchor on development and long-term recovery.

## The study reported in this paper

In this paper, we examine the question of the nature of informal support-seeking and provision among a sample of people who were present at the Manchester Arena bombing in May 2017, but who were not physically injured. Our focus is on what survivors told us about their experiences of, mainly, informal or horizontal social support. Our other paper, which is part of the same project, explores the distress experienced by, and opinions of, people about state-funded vertical support services.^13 cf 35^

In the interviews, we addressed three questions. First, what is the nature of any informal or horizontal support-seeking and provision for some people who experienced the Manchester Arena terrorist attack? Second, who provided that support, and what makes it helpful? Third, to what extent does being part of a support group based on shared experience of the attack operate as a springboard to recovery?

In the aftermath of the bombing, the Manchester Resilience Hub (the Hub) was established to support adults, children and professionals experiencing distress and refer survivors who required it to other services, including specialist mental healthcare. Within the first year after the attack, the Hub registered 3150 people, representing 16.2% of all those who were physically present at the attack.^1^ The purpose of this paper is to understand how informal support was experienced following that emergency or major incident. Our previous paper explores participants’ perceptions of the more formal, vertical support facilitated by the Hub.^13^ Together, the two papers provide practical recommendations on how beneficial forms of support can be facilitated and assist the similar centres set up after future events.

It is important to note that the Hub was not the only formal structure set up after the Manchester Arena attack. Also, there was sometimes a connection between formal or vertical and informal or horizontal support. For example, a Manchester Attack Support Group Programme was set up to support the people affected.^3,46^ It organised peer support groups for bereaved people, and also provided emotional support and signposting to other services. Most of the people who joined the support groups were survivors, but bereaved people and responders also participated, with people attending from different areas of North England and beyond (i.e. not solely Greater Manchester). These groups were run by experienced facilitators, but were a way by which survivors and bereaved could meet others like themselves. Also, there was a variety of other support groups and networks, including the Manchester Survivors Choir and Survivors Against Terror. There were also a number of citywide solidarity initiatives, including those promoting the Manchester identity via the symbol of the worker bee, which was displayed on many buildings around the city in the months afterward ‘to represent Manchester's indomitable spirit’.^47^ Additionally, there were several horizontal online networks and groups set up by survivors.

Our participants were 18 people registered at the Hub. Everyone registered at the Hub had undertaken online assessments as a form of screening at 3 or 6 months after the attack. To explore possible differences in their experiences of support relating to the severity of their scores, our sample comprised seven who were categorised as having a severe reaction, six as a moderate reaction and five as a mild reaction. Semi-structured interviews enabled us to probe responses and examine in depth the quality of experiences with family members, friends and peer groups.

We believe that our research questions have not been addressed previously in the Manchester Arena case, although we acknowledge that similar matters have been examined in relation to other terrorist events.^46^ Based on the previous literature, we expected families, friends and valued groups to be important as sources of expected support. We also expected that shared experience of the incident would be important to participants in their accounts of effective support. Therefore, we hypothesised that groups and networks that arose from the Manchester Arena incident would provide important forms of support through their shared experience, which can provide a way in which people feel able to disclose safely and feel understood.^3^

## Method

### Participants

Interviewees were 18 registrants with the NHS Manchester Resilience Hub. All registrants at the Hub were invited to indicate whether they would like to participate in future research, and this created a list of Hub users (*n* = 262) from which the sample was drawn. A purposive diversity sample was constructed from this list on the basis of the scores of eligible persons on the Manchester Hub online measures that included: the Trauma Screening Questionnaire (TSQ),^48^ the Patient Health Questionnaire-9 (PHQ-9),^49^ the Generalised Anxiety Disorder-7 (GAD-7)^50^ and the Work and Social Adjustment Scale (WSAS).^51^ All these measures are standardised and validated with established clinical cut-off points. The criteria used by the Hub to assign clinical priority at triage are based on registrants’ initial scores on these psychological questionnaires.

We sought to recruit participants evenly split across three subgroups who showed responses on the measures used routinely by the Hub that were consistent with one of the three broad patterns (‘mild’, ‘moderate’ and ‘severe’ reactions) in which people respond to emergencies and incidents.^11,15,52^ This was intended to enable us to explore whether experiences of support differed across levels of distress response. The three subgroups were operationalised as follows:

- mild reaction: TSQ score <6, PHQ-9 score 0–9, GAD-7 score 5–9 and WSAS score 1–10 (seven participants).
- moderate reaction: TSQ score of 6; and/or PHQ-9 score of 10–19 or a score of 1 on the self-harm item; and/or GAD-7 score 10–14 and/or WSAS score 11–20 (six participants).
- severe reaction: TSQ score ≥6 and/or PHQ-9 score 20–27 or scores ≥2 on the self-harm item; and/or GAD-7 score ≥15; and/or WSAS score ≥21 (five participants).

The interviews were conducted over a 4-month period, between October 2019 and January 2020, and participants were asked about their experiences at the time of the bombing and in the intervening period.

### Eligibility criteria

Eligible participants were defined as those who met the following criteria: they had attended the concert at the Manchester Arena in May 2017, but had not been physically injured by the bomb; they had at least one assessment on the Hub's psychometric screening measures at the 3- and 6-month post-event time points; they were aged ≥18 years at the date of their initial assessment and they were Hub users who had given consent to be contacted about participation in evaluation and research.

In addition, the researchers endeavoured to ensure the sample contained people who had a spread of home addresses (i.e. addresses inside and outside Greater Manchester), and ensure that parents and young people were represented. Mean average age was 33.4 years (range: 18–55 years). Nine participants were parents. Seventeen participants were female.

### Interview schedule

The interview was semi-structured and organised around the following topic areas: (a) the social context before the event (e.g., ‘How would you describe what life was like for you before the Arena event?’); (b) experience at the event and immediately after (e.g. ‘Going in to as much detail as you feel comfortable with, what did you experience at the Arena that night?’); and (c) social influences on coping and recovery (e.g. ‘Looking back, who or what has helped you cope or recover from the event?’ ‘Is there anyone or anything that has hindered you in your coping and recovery?’). Some of the interview topics including, in particular, experiences of distress and official support services, are not covered in this paper.^13^ The full interview schedule is in the Supplementary Material available at https://doi.org/10.1192/bjo.2022.528.

### Conduct of the interviews

Each of the semi-structured interviews was conducted by one of two of the authors by telephone. Each interview lasted up to 1 h. Each participant was offered to have a person of their choice present to support them and follow-up support. Just one person took up the offer of support. Each interview was recorded and was transcribed verbatim.

### Analysis

The interviews were analysed with thematic analysis.^53^ The approach to thematic analysis was both theory-driven and inductive. For example, based on previous research, we were particularly interested in participants’ accounts of the support provided by families, friends and groups they felt they were psychologically part of; however, we were particularly interested to identify any experiences of importance to participants, within the broad question of support after the incident, but which were not known to us *a priori*. Each transcript was read by five of the authors including the two interviewers, and a number of issues that seemed important to interviewees were coded (e.g. the variety of experiences of support). Each transcript was then subjected to detailed thematic analysis. Three of the authors independently coded and developed suggestions on themes before coming together to compare their definitions and to merge and split themes where appropriate. Through this iterative process, a thematic structure was agreed. Within the overall structure, there were themes around experiences of different types of support (e.g. ‘social validation’) and of being part of support groups (e.g. ‘shared experience as group identity’), plus many other themes not used in the present analysis (e.g. experiences of distress and services offering psychosocial care).^13^ The agreed thematic structure was tabulated with definitions and examples, for easy visual inspection, and its reliability further checked by asking someone outside the project to apply it with a subsample of the data.

### Ethics statement

The authors assert that all procedures contributing to this work comply with the ethical standards of the relevant national and institutional committees on human experimentation and with the Helsinki Declaration of 1975, as revised in 2008. All procedures involving human patients were approved by the UK's Integrated Research Application System (IRAS) process (application 255819). Written informed consent was obtained from all participants.

## Results

Our results are based on the information offered by our participants in response to open-ended questions augmented by prompts. We were keen to enable participants to share their experiences and perceptions, and endeavoured to view matters from the participants’ own perspectives. We do not attempt to provide a full account or categorisation of the different effects of all the services and support facilitated by the Hub and other agencies. Consequently, our analysis focuses on interviewees’ accounts of sharing their emotional concerns with others, constraints on seeking support, experiences of support, being part of informal support groups and support group dynamics. The overall thematic structure for the study is shown in Table 1. We selected extracts for presentation to represent each theme.

**Table 1:** Thematic structure

Superordinate themes	Themes
	Sharing emotional concerns
Constraints on seeking support	Internal
	External
Experiences of support	Social validation
	Emotional support
	Instrumental/material support
	Informational support
	Giving support to others
	Knowing support is available
Being part of support groups	Shared experience as group identity
	Actions to create or maintain connection with group
	Lack of identification with others
Support group dynamics	Too much focus on trauma
	New friendships
	New support
	Personal growth

### Sharing emotional concerns with other people

Our interviewees said that, in the aftermath of the attack, they had a variety of distressing experiences, including guilt, shame, anger, anxiety, dissociation, intrusive thoughts, a sense of insecurity and moral distress.^13^ They said they wanted to share their emotional concerns with others. Twelve of them described experiences as in extract 1, in which their desire to share experiences was based on their beliefs that it would help reduce their distress:

Extract 1

‘I have spoken to people who have had PTSD in the past and I knew that I had to talk about it and I knew what I had to do in my mind because I am an intelligent woman, so if anybody was prepared to listen, I just told them the story. I know I gave you the short version but the story of what happened that night, I told everybody it because I knew that the more you tell it the less upset you get.’ Participant 16 (mild).

Most interviewees (16 out of 18) reported emotional sharing with friends and families in the immediate aftermath of the attack. This pattern was similar across the three subgroups. Eleven of these 16 participants also said they shared emotionally with others who had experienced the attack or similar attacks. As we shall see, there were often important differences between these two potential sources of support. One difference was in terms of how interviewees accessed people to share. In particular, some of this disclosure was in person, but some had to be online, if that was how they could find others who had experienced the attack:

Extract 2

Participant 5: Well, I spoke to my sister and my sister's girlfriend like every day because I was with them. At the time, it felt like I could only really speak about what happened with them.

Interviewer: Yes, yes … Was that because they'd sort of shared … yeah.

Participant 5: And I guess if I didn't have them, I might have been more likely to speak to someone online that was there.

Participant 5 (moderate).

### Constraints on seeking support

The interviewee quoted in extract 1 referred to telling ‘everybody’ about what happened. Two others explicitly reported disclosing freely: one (participant 5; ‘moderate’) had friends and family at the arena; the other (participant 6; ‘mild’) reported low levels of distress and shared with friends via social media. However, most of our interviewees (*n* = 16) reported reluctance to talk about their experiences and feelings or to seek support. For these interviewees, the degree of emotional sharing, in terms of both quality and quantity, appears to have been moderated by what we have denoted ‘intrinsic’ and ‘extrinsic’ constraints. We categorised ‘intrinsic’ forms of constraint as those examples in which interviewees referred to their own feelings of reluctance to initiate sharing or talk about experience of distress with others. This was reported by 13 interviewees. More participants who scored as ‘mild’ on the psychological measures (six out of seven) reported intrinsic constraints compared with those with ‘severe’ scores (four out five) and ‘moderate’ scores (three out of six). The most common reason given for intrinsic constraint was not wanting to burden and/or upset friends and family members (11 participants). This was followed in order of frequency by ‘family and friends wouldn't understand’ (*n* = 3), ‘too difficult or emotive to talk about’ (*n* = 2), the interviewee being (what they referred to as) ‘in denial’ (*n* = 1) and being ‘undeserving’ (*n* = 1).

For example, participant 8 (extract 3) referred to their perceived ‘burden’ to others, implying that their own concerns and feelings are less important than those of others, as well as the idea that expressing these feelings is ‘weak’:

Extract 3

‘You know I don't like being a burden, I am quite self-sufficient and independent and you know I don't like that whole showing signs of weakness, you know if you're upset in front of people and all that … even though it's really not a good thing to do that's how I am as a person and I kind of just bottle it up and that didn't do me any favours in the beginning … but I wouldn't change things, if it happened again I wouldn't burden people again with how I feel because people have got their own things going on haven't they.’ Participant 8 (severe).

We categorise ‘extrinsic’ examples of constraint as those in which interviewees expressed a reluctance to share or talk about experiences with others following an actual or perceived negative experience of sharing, based on the reaction of the person with whom they were sharing. This was reported by nine participants, relatively evenly spread across the subgroups (‘moderate’ four out of six, ‘severe’ three out of five, and ‘mild’ two out of seven). The most commonly reported experience was of some form of social invalidation by friends and families; for example, shutting down the conversation, being awkward, stepping back and responding inappropriately. In the following example of awkwardness, the close family member was willing but apparently unable to provide the support needed:

Extract 4

‘Mum didn't know what to say from what I can remember, the main support I had was from my mum, because I think a lot of my family were just like “Oh I'm really glad you're okay” whereas my mum was like piecing together that I was okay, like I was alive and I survived but I wasn't okay … so I think in terms of actual support it was mainly from my mum … and I think even then like my mum didn't really know what to say, I didn't really know what to say to her … like even then it was quite strange because neither of us really knew what to say to each other.’ Participant 15 (moderate).

For a smaller number – three of the people who reported ‘extrinsic’ constraints (one in the ‘severe’ group and two from the ‘moderate’ group) – there was emotional upset or conflict with friends or family members in such encounters. Some reported that their families or friends did not understand their experience. In the example in extract 5, expressing this feeling of not being understood led to anger and upset on the part of the other person:

Extract 5

‘I just felt like people didn't understand what I was saying, so I kind of stopped talking about it, because they didn't understand … I remember saying to one of them that I hadn't been sleeping very well after a few weeks and whatever and she was asking how I was and I just kind of said … “well I'm not even talking about it anymore because nobody even understands”, and she was kind of like “what do you mean no one understands, how dare you say something like that”.’ Participant 8 (severe).

Therefore, how partners, close friends and family members responded to emotional disclosure of distress affected the likelihood of further sharing.

### Experiences of forms of social support

Interviewees mentioned a variety of types of informal social support that they found helpful. In examples of emotional support, participants described interactions that fostered feelings of comfort leading them to believe that they were loved, respected and/or cared for by others. Participant 6, for example, described an experience at work:

Extract 6

Interviewer: Yeah … and when those thoughts pop up, do you feel like anything helps you in those situations?

Participant 6: Maybe just messaging my friend or something … and they'll just say ‘no don't worry, it will just be this, just be that’.

Interviewer: Yeah … and does that help?

Participant 6: Yeah.

Participant 6 (moderate).

Informational support refers to relevant information, advice or guidance that the interviewee said helped them cope with current difficulties or understand the event:

Extract 7

Interviewer: Okay … and you mentioned about your uncle giving you a call as well, did you feel like that was helpful the information that he had given to you?

Participant 13: Yeah, it was … considering he had been through it before … so it was helpful to know what was going to happen and at that point not that much had been revealed it was all just speculation, so it was like “okay so we know this is going to happen” … and that was good.

Participant 13 (moderate).

Instrumental/material support refers to experiences of goods and services that helped the interviewee solve problems or assisted coping:

Extract 8

‘I was struggling to eat after, my mum was trying to get me to eat again but I just didn't really have an appetite. For the couple of day - like straight after - I struggled to get out of bed so … I think just looking back now it was like … like my mum even got me out of bed … and for the first day that was like progress.’ Participant 5 (moderate).

Giving support to others refers to the experience or perception that they have been helpful to others, in terms of meeting emotional or instrumental needs. This extract reports the motivations of participant 7 for setting up an informal horizontal group:

Extract 9

‘So, I go to one of the meet ups and realise for me and my daughter this is absolutely invaluable … so, I came home and decided that I wanted to make a different to help the kids … because I could see how much the kids were suffering and I wanted to do something regularly, not just intermittently around the UK … I wanted a regular thing … so I found a venue, I set it all up … I had lots and lots of meetings.’ Participant 7 (severe).

Knowing support is available, similar to expected^54^ or perceived support,^20^ is the knowledge or perception that people or services are available and accessible if and when needed:

Extract 10

‘I had messages off everyone I worked with … my mum lives in [city] so she was ringing me and my aunties and stuff that live there … my dad had rang me, I can't remember where he was working at the time but he was ringing me … and it was just nice to know that there was people there.’ Participant 2 (moderate).

Social validation was particularly important to interviewees. This refers to participants saying that their emotional concerns and experiences of distress had been heard, understood and acknowledged by others. It was the form of helpful support mentioned by more interviewees than any other; 12 out of the 18 participants (four from each subgroup) described these experiences. In extract 11, for example, participant 8 described how her husband validated her feelings by respecting and acknowledging them, thereby taking her seriously:

Extract 11

‘He [husband] would never undermine the way that I'm feeling with stupid comments … so he was great … I think he [husband] just understood me … let me talk to him … he never made me feel bad about the way that I felt.’ Participant 8 (severe).

Crucially, this understanding was linked in participants’ accounts to unconditional acceptance and listening. This experience of validation as specifically being understood was something that six other interviewees also mentioned.

Many of the interviewees’ comments suggest that the source was important in these experiences of social validation; validation seemed to be experienced as more helpful when provided from some sources rather than others. Nine participants reported experiencing social validation by family members and eight by friends. However just over half of these examples (nine of the 17 reports of social validation spread evenly across the subgroups) referred to social validation from friends or family members who had common experience of the attack. In the following example of validation with others who had also experienced the attack, disclosure led to the recognition of similar feelings, which was helpful in itself, but also enabled reassurance:

Extract 12

Interviewer: Yes, yes. So, with your sister and her girlfriend, what kind of things would you speak with them about that helped?

Participant 5: Erm - mostly about sort of how we were feeling and when we were speaking, we realised that we were all feeling the same, and this then made us feel better and it wasn't just … we were just sort of just telling each other it was OK?

Participant 5 (moderate).

Participants reported mixed experiences of the support offered by those friends and family members who did not have a common experience of the attack. Here, social invalidation was more frequently reported than social validation. In these cases, emotional disclosure was rejected, ignored or judged. For example:

Extract 13

‘My dad doesn't do well with like emotional stuff, he was just like “you'll be fine buddy, just get on with it”, that's the kind of message he gives.' Participant 13 (moderate).

Thirteen participants (‘severe’ four out of five; ‘moderate’ four out of six; and ‘mild’ five out of seven) described experiences of social invalidation from families and/or friends. All were with people who did not experience the attack. As we have seen earlier, this invalidation led to reluctance to talk. Therefore, although social validation was prominent in accounts of helpful social support experiences, invalidation seemed to be important in the accounts of unhelpful experiences.

### Being a member of support groups

In the interviewees’ accounts, social groups comprising people who had in common experiences of distressing major incidents they described as ‘trauma’ were a key source of social support. One interviewee (participant 8) talked about a face-to-face group that comprised people from different traumatic events. In all probability, this was a multidimensional group. Other participants were involved with groups that included people affected by the Arena bombing. Of these, two interviewees (participants 1 and 7) were in online groups that comprised solely Arena survivors to start with, but later included survivors of other attacks (e.g. the Tunisia and London Westminster Bridge incidents) joined. Both thought that this mixed composition had a negative impact, and both decided to leave. Although it is unclear what the origins of these groups were, the context indicates that some were horizontal in nature and others were vertical.

Fifteen participants stressed the importance of joining a group of people who had shared experiences. Interviewees engaged with these groups both face-to-face and online. Ten participants, evenly spread across the severity subgroups, described experiences only with face-to-face groups, four with online groups only and one with both face-to-face and online groups.

Of the three people who did not join such groups, participant 2 (‘mild’) had attended the Arena event with two friends and one of them became an important source of social support; participant 3 (‘mild’) saw psychological distance from Manchester as helpful; and participant 9 (‘severe’) was socially withdrawn and avoided social media.

Although the interviews do not address the origins of each of these groups, some were organised by the Hub (vertical groups) and it is clear that the psychological processes involved in participants joining and engaging with support groups were different from those of support-seeking with friends and families, on a number of levels. First, interviewees engaged with the groups not because of who the members were personally but because, as a group, they were similar to self on an important dimension (common experience of the attack). This is evident in the following example, which refers to similarity to self (in terms of the Arena experience) as the key quality of group members:

Extract 14

‘… and it was genuinely changed for me because you suddenly realised - hang on a second I wasn't on my own - and I think that in itself was a real, really helpful for me … it was the first time I'd ever met anyone that was in the same situation as myself … and then in the last year they [Hub] set up support groups so I go to the support groups every month … wonderful to meet people in a similar situation and that I think that's really helping my recovery.’ Participant 10 (severe).

Or, more succinctly:

Extract 15

‘It's the link that binds us.’ Participant 4 (mild).

In other words, there was a sense of ‘us’ – or identification – from being part of these groups based on shared experience.

Second, engagement with these groups came after support-seeking with friends and family members, and often occurred because interviewees were not getting sufficient support from those close to them. Third, some participants told us that their engagement was actively sought and maintained through deliberate actions, such as carrying out online searches or attending organised events. For example, participant 18 describes joining online groups to find others:

Extract 16

‘So, it was good to know that there were other people on, like, Instagram and Facebook that were there and related to it. My friend [name of friend with whom she went to the concert] was affected differently so I found the people that it affected in a similar way …’ Participant 18 (mild).

Thus, participant 18 expressed substantial agency in finding the support they desired. Similarly, other participants described their actions to actively create or maintain connections with a wider Manchester solidarity community, which was unrelated to the Hub, such as getting a bee tattoo and having memorial pictures:

Extract 17

Participant 5: … and then I think it was the week after I got the Manchester bee tattoo … it actually helped me a lot more than I thought.

Interviewer: Yes?

Participant 5: Yeah, yeah. It kind of felt like I was putting something good back into it. Like the money went towards the charity and it felt like we were part of something better. Yes, she [sister] was sending me like all of the memorials and stuff. It kind of made me feel better that they had gone back to Manchester and were, like, enjoying being in the community.

Participant 5 (moderate).

Twelve interviewees, evenly spread across the subgroups, took deliberate action to create or maintain connection with support groups online or face-to-face (four online only, four face-to-face only and four both). Thus, even when groups were initiated vertically, our participants report expressing substantial agency in deciding which to join. The groups chosen tended to allow unfettered emotional disclosure free from the intrinsic and extrinsic constraints associated with friends and family. Hence, the groups provided validation and other forms of support in a way that other relationships did not. This was experienced as very helpful:

Extract 18

‘… hen it happened, I didn't know anybody to talk to that was there who might understand … so I need people that were like minded, so we've got a group of us, we meet every few months in Liverpool and we have a WhatsApp group and that's just a tremendous help.’ Participant 16 (mild).

In fact, participants sometimes explicitly contrasted their openness in disclosure and the understanding and acceptance they received in groups with their experiences with friends and family. For example, participant 12 explained that their family wanted to help, but being unable to do so is distressing for them:

Extract 19

‘… I think you have got to have been there to understand, which is why that focus group was really good, because they all said the same … family are sympathetic and they're there for you and they will do whatever you want them to do, and if you're upset they will comfort you and things like that but they get frustrated because they don't understand how you feel or why you feel like you do …’ Participant 12 (moderate).

Participants described feeling a sense of isolation, inability to make sense of their distress and inherent threats to self-worth before they made active contact with other people who were at the Arena. After engagement with a support group, they said they appreciated that they were ‘not alone’; their experience of distress was appropriate and normal, and thus not a sign of personal weakness or inadequacy:

Extract 20

‘Yeah, like, I say, them groups were really good … like I say … if anything, if you had to just pick one thing and say well you can only have one thing you can't have all the others that go with it … that would be the one, because you are with people who understand what you're feeling and don't think you're being silly.’ Participant 12 (moderate).

### Support group dynamics

For three interviewees, being part of a support group crystalised a sense of collective identity as ‘those who were psychologically injured in the attack’, which was contrasted with those who were physically injured, whose needs they felt were more recognised and catered for. This lack of recognition by others was associated with a sense of resentment:

Extract 21

‘I just get so cross with how … and this sounds awful … but the people who lost family or was injured, they've been sort of included into this inner sanctum thing and the rest of us was just all forgotten about … when it came to the first year anniversary, we went to Liverpool Cathedral and there were some people there who had been hurt or whatever and they all knew each other and I was sort of sitting going well how do they all know each other? … why don't we know them? We knew some were from [city where interviewee lives] so it was really frustrating that we didn't get help quicker … so it's been us outside, you know, outside the inner sanctum, we haven't really had help I don't feel.’ Participant 16 (mild).

For the other two participants (participant 1 (moderate) and participant 4 (mild)), the identity of the group as one defined by the attack meant that they worried that the group dwelled too much on their ‘trauma’:

Extract 22

‘I think it's good that we talk about it, but then sometimes we feel … I don't know if sometimes we talk too much about it, you see.’ Participant 4 (mild).

However, interviewees were overwhelmingly positive about the sharing opportunities offered by the groups they were part of. Being able to disclose and hear from others in the group who were present at the attack had benefits beyond the subjective sense of not feeling alone. Indeed, for some, there were tangible changes in their relationships beyond the group itself. Thus, for some, the connection created by the group was the basis of new personal friendships:

Extract 23

‘Them focus groups … they were really good because … you just felt like you weren't going mad, because that's what you start to think, I'm going mad … and other people would be like no because I feel like that as well and I thought I was going mad … they were so useful … I'm still in touch with 2 of them that went now, and we meet up now and again for a coffee and the children come and they get on as well … so, yeah, that was really, really good.’ Participant 12 (moderate).

Some interviewees said that active engagement and relationships in the group opened up access not only to further validation but also other mechanisms of social support, as provider as well as recipient:

Extract 24

‘There was two people I actually met from the workshop last year, so I still keep in contact with them … so yeah, I have me people through this experience and you know I can talk to them, and they can talk to me … not compare our experiences, but kind of help give each other ways to help each other cope.’ Participant 11 (severe).

There is evidence that being part of a group did not appear to mean becoming anchored in an unhelpful circular focus on the attack and their distress. Thus, some participants said that they were able to use being part of the group to make positive changes in their internal or external world beyond the group. For example, participant 10 described how group members provided practical support in the form of advice for ‘troubles’ beyond the Arena experience:

Extract 25

Interviewer: So, what kind of things do you do in the support group?

Participant 10: We honestly just talk about stuff we feel we want to talk about and that doesn't have to be arena related, sometimes it can be troubles that people are having … we tend to share advice or kind or what's another word? Like tips on how to help or suggestions on things to do …

Participant 10 (severe).

She also described taking up a new activity, yoga, which she attributed to being part of the group:

Extract 26

‘I'm definitely recovering … and like I said things like the support group started, I started going to yoga, I started doing things that were important to me, you could kind of deal with it more privately, I think.’ Participant 10 (severe).

For 11 of the interviewees (evenly spread across the subgroups), the support provided by the group was formative of personal growth. The support resulted in less need for active engagement with the group over time, with the group, in effect, operating as a springboard for recovery in the form of life beyond the Arena experience and beyond the group. Interviewees reported this experience in themselves and observed it in others:

Extract 27

‘Yeah, I had a good group … we ended up having a meet up in the June, I think it was, … but yeah, they have been a massive support to me … I give myself more credit than I would of before … you know I don't view myself as a weak person anymore … I just think I'm back to normal, I think I view myself, how I should view myself now.’ Participant 8 (severe).

## Discussion

### Sharing emotional concerns with other people

Survivors of the Manchester Arena bombing attack in 2017 who were interviewed in our study experienced significant distress after the event and sought to discuss their feelings with others. Most survivors turned to their families and friends for comfort in the immediate aftermath, which was more likely to be helpful if friends or family members shared their experiences.

All but two of our participants also reported reluctance to talk to others. On one hand, this reluctance stemmed from their own concerns with burdening or upsetting them, beliefs that others would not understand or feeling that it would be too difficult to talk about. On the other hand, the reactions of other people that survivors observed when they did try to disclose their experiences were a further source of reluctance. They described experiencing social invalidation by others – in particular friends and family members who were not at the incident would, for example, shut down the conversation, or were awkward, avoidant or responded inappropriately.

### What was the nature of informal social support for survivors of the Manchester Arena bombing?

Interviewees described a variety of informal support mechanisms that they found helpful, including emotional, informational and practical/instrumental support; social validation; providing support for others; and perceiving that support was available. Emotional support and the related social validation were more common than practical and informational support in the reported experiences of our interviewees. These different experiences of support were reported across the subgroups (mild, moderate and severe reaction).

A number of large-scale surveys of experiences of support, including panel studies of coping and recovery after disasters, show that it is the perception or expectation of social support, more than the frequency of actual social supportive action, which has beneficial effects on well-being.^5,17,20^ We have observed this and the importance of validation anecdotally but recurrently when working with staff of the NHS during the pandemic. For our interviewees, experiences in which support was expected but not received led to distress on top of the distress based on the bombing. On some occasions, failure to get the expected support led to interactional trouble and conflict. These experiences applied to friends and family members – the people to whom interviewees were close, but who did not understand their needs, or were unable to meet them. This finding resonates with that of Dyregrov et al,^30^ where bereaved parents of victims of the Utøya attack reported that inept support, in the form of poor interaction uncalibrated to their needs, was very stressful in their support-seeking experiences.

Our finding that social validation was particularly important tallies with research which shows that bereaved parents of victims of the Utøya attack were most helped by ‘mutual understanding’ with other parents with shared experience.^30^ Our finding that being understood was a crucial component of validation is in line with new experimental research showing that feeling understood is a fundamental mechanism in the ‘social cure’.^55^ However, although our evidence suggests that shared experience was crucial overall, it was not always necessary for being understood. For example, in extract 11, participant 8 said her husband (who was not with her at the Arena) was understanding in a helpful way. In addition, some interviewees talked about validation, including being understood, from mental health professionals. We report similar perceptions with regard to the services provided by the Hub in our previous paper.^13^

### Who provided support, and what makes it helpful?

Consideration of the nature of support takes us to a fuller discussion of the complex role of shared experience. Support from others like oneself is increasingly recognised as a key part of the recovery process following collective tragedies.^3,32,35^ In our interviews, as Harms et al^56^ found in relation to the Australian bushfires, families and friends can act as a source of support particularly when they have shared experience of the distressing incident. As has also been found in previous research,^31^ our analysis suggests that people who did *not* share experience of the attack, including families and friends, may find it more difficult to provide the support that survivors need; families and friends may try and fail, or they might avoid trying. By contrast, there were numerous reports of effective support being provided by groups (i.e. comprising people who were not friends or family members), whether of vertical or horizontal origins, defined in terms of shared experience. This observation links to a broader point about support: that it is most effective when providers and recipients share the same framework for interpreting it, via sharing the same social identity.^38^ By the same token, where a group resists the aid offered by another group after disasters or conflicts it may be because the two groups have very different interests in the aid-giving relationship (e.g. dominance versus dependency).^57^

The role of psychological membership of groups in well-being is now well-established both for everyday group memberships^38^ and for people who define themselves as a group through their common experience of a major incident.^41,43^ But not all group memberships associated with extreme events are good for mental health.^44^ Therefore, it is important to understand the psychological conditions under which informal support does or does not assist people to recover.

### To what extent do support groups based on shared experience of the attack operate as a springboard to recovery?

There is an argument that groups and identities focused on the shared ‘trauma’ can hold back recovery, by crowding out other identities, and by preventing support from other people who do not have the shared experience. Among our interviewees, there was some recognition of this possibility. Participant 3 saw psychological distance from Manchester as helpful. Participants 1 and 4 worried that their support group talked too much about their common distressing experiences. Yet, both of these interviewees also described benefits of being part of the group. Indeed, our analysis did not find evidence that being part of a group based on shared experience served to cut interviewees off from other sources of support – quite the opposite. First, at least in some cases, the temporal sequence was the other way round; reaching the limitations of support from family members contributed to people seeking out other survivors in the form of vertical and horizontal support groups. Second, and more importantly, most participants said that they were able to use being part of these support groups to make positive changes in their internal or external world beyond the group: their friendships went beyond the support group, and they developed new interests and a new sense of self. Some of our participants made clear their active engagement in choosing among groups to gain what they saw as important. These groups acted as a springboard, not an anchor on recovery.^58^

This is not a claim that support groups always aid full recovery. There might be particular features of groups in which survivors of the Manchester Arena attack engaged that aided personal growth; or it might be that other survivors not in our sample had different experiences than did our interviewees. But in this case, as in others,^59^ on balance, it seems that strategic efforts to maintain groups (e.g. by organising meetings, having a group identity, maintaining the network) months or more after the incident can be beneficial.

It is important to acknowledge the sheer variety of informal or emergent support groups of which our participants reported experience. They ranged from online groups to face-to-face (or, more properly, ‘in-person’) groups, to wider networks. An example of the latter is participant 5 getting a tattoo of the Manchester worker bee to connect with the wider Manchester solidarity community. Some of the groups were horizontal in nature, others were vertical, and thus closer to our notion of being formal, whereas others were multidimensional.

We did not detect differences in the benefits people obtained from online compared with in-person groups. Certain risks associated with broad solidarity campaigns (compared with small in-person groups) were apparent, however. The very openness of some groups meant that many different people could get involved, which was not always welcome. A number of our participants did not identify with groups that were defined too broadly as related to a variety of different incidents, indicating, we think, that the context offered by groups is important to people who join them. In these instances, we conjecture that the gains from group identity were insufficient to justify the trade-offs required to share lived experience of as diverse set of incidents. This resonates with views proposed by Muldoon and Lowe.^60^ Another example is provided by one of our interviewees who described those people identifying through their adoption of symbols with the wider Manchester identity as ‘bandwagon jumping’ (participant 17), meaning inauthentic and not really engaging with the needs of people who were affected. In addition, comments might not be filtered in groups operating via social media. Some interviewees found comments by others on social media distressing, even if those comments were well intended. The distress caused to survivors and bereaved by intrusive media has been noted following other recent terrorist attacks.^46^ These risks of unconditional or unbounded sharing of traumatic details and distress having negative outcomes for ‘listeners’ need to be examined more systematically, and the stated benefits of these groups need to be weighed against these risks.

Another feature of the wider solidarity campaigns that some interviewees felt was not helpful for their own progress was the memorial events and commemorations. Although some found these occasions helpful as ways of connecting with others, some interviewees found them unhelpful as they were reminders of their distress, of which they were not in control. On this point about commemorations, it can be instructive to compare experiences of participants in the Manchester Arena bombing with some of those in the case of another tragedy that took place only a few months later, the Grenfell Tower fire, in which 72 people were killed. Unlike Manchester, where the people affected were geographically dispersed, Grenfell was a local community-based tragedy. Survivors, bereaved people and their local supporters organised themselves to exert some control over the broader solidarity campaign. They also held their own commemoration every month.^61^ In this case, the people affected were the ones in control of the commemorations.

### Strengths and limitations

A strength of the present study design is the depth and quality of the data and the richness of the accounts of our participants. A weakness, as withall studies of survivors of disasters, is the fact that the participants are only those willing to talk. Those people who were unwilling to talk, or who did not even connect with the Hub, may have different experiences.

It is a strength of the design that the sample composition was a function of careful psychometric testing to enable us to gauge the severity of their experiences or responses of a range of participants. The findings we present here are one part of a larger programme of work on distress, support, coping and recovery among the people affected by the Manchester Arena bombing.^13^ Other kinds of research evidence are needed, including survey data to measure prevalence of some of the experiences analysed here.

Finally, this study was not designed to examine the reasons for possible differences in effectiveness of the various forms of informal social support. We did not seek to systematically examine different effects of horizontal versus other forms of support because participants themselves did not always clearly distinguish between them. In addition, some participants engaged with multiple forms of support, and some were able to access some forms, but not others. For example, one interviewee living outside Manchester mentioned how the local solidarity campaign gave him comfort; in this case, his sister sent him materials to keep him in touch and make him feel connected. In other cases, it is possible that the wider solidarity campaign was invisible. Yet others may not have been able to attend in-person group meetings or access online groups through digital exclusion.

### Practical implications

Given the social, mental health and experiential significance of terrorist attacks, there is a theoretical and practical need to understand the role of informal as well as formal social support. In this study, we have seen that validation is important to survivors and that there are risks resulting from invalidation. Organisers of services that aim to support survivors should be helped to understand what it is that survivors seek in the immediate aftermath from their families and friends, so that family members can be supported in meeting the needs of their relatives effectively.

We have found important evidence that informal social support, and in particular that provided by groups based on shared experience, can be psychologically beneficial to participants. This suggests that this kind of support helps people to cope and aids their recovery, as has been shown in research on other terrorist attacks.^28^

Two further points about recovery are important. First, the concept of ‘recovery’ after disasters for survivors is complex. Our other research on survivors of the Manchester Arena bombing suggests that some continued to experience serious distress 36 months after the incident.^13^ Second, beyond recovery narrowly understood in individual terms, informal support groups can benefit the group of survivors and bereaved people as a whole as well as the wider communities of which they are a part. This is because they can serve as organising bodies for campaigning for improved compensation, improved services, legal change and justice.^3^ This has been witnessed after Aberfan, Hillsborough and Grenfell.^61^ This means, in turn, that it is important to consider how support groups can themselves be supported.

The Manchester Attack Support Group Programme is one model for support, but there are many ways of achieving the same thing. Broadly, there is a need to proactively promote social connectedness. Some interviewees described problems in finding other people with whom to share experiences and they felt a sense of isolation until making these connections. Therefore, facilitating access to groups of survivors should be initiated as early as possible. The Manchester Hub provided all registrants with details of formal support group offers, as did a dedicated website provided by the local authority. Such networking support should be informed by what is known about the nature of psychosocial care. This means that the care pathways offered should be broader than solely including links between and with clinical and social services, and include making people aware of support groups for survivors. In addition, resources for these groups should be provided, such as making available spaces for in-person meetings and access to free Zoom accounts.

In conclusion, we have learned from our participants a variety of features of social support that they found helpful and some that they did not. A most important finding relates to the importance of validation and the negative perceived impacts of invalidating people's lived experiences.

Terrorist attacks cause widespread distress, as well as mental ill health for smaller numbers of survivors. Much of the psychosocial care that affected people receive is informal and is provided by families, friends, social networks and horizontal peer groups. Group support can promote well-being,^38^ but can also assist those people struggling with distress and mental health problems after major terrorist incidents.^11^ Vertically organised and facilitated formal groups are also highly important, but have differing, if overlapping functions.

Another key matter shown in some of our participants’ accounts is that they express their own preferences in choosing which groups to join. Care is needed to balance the desires and knowledge of professionals with survivors’ preferences.

Incidents such as terrorist attacks have increased in recent years. There is a need to better understand and facilitate the informal social support given to survivors. Our findings confirm previous research on the experience of survivors, showing the usefulness of informal support after disasters and major incidents also applies in the case of survivors of the Manchester Arena attack. In addition, we have shown that benefits of in-person groups based on shared experience also apply to online groups and wider social networks.

We recommend that organisations that respond to people's psychosocial and mental healthcare needs after disasters and major incidents should resource support groups and more generally facilitate connectedness between survivors. People who respond to survivors’ psychosocial and mental healthcare needs after emergencies and major incidents should facilitate interventions for survivors and their social networks that maximise the benefits of social validation and shared experience, and minimise the harm associated with social invalidation.

## Data Availability

The data are not publicly available due to their containing information that could compromise the privacy of research participants.

## Supplementary material

For supplementary material accompanying this paper visit http://doi.org/10.1192/bjo.2022.528. click here to view supplementary material
