# Supplementary material for: Survivors’ experiences of informal social support in coping and recovering after the 2017 Manchester Arena bombing
Source: BJPsych Open. 2022 Jul 4;8(4):e124. doi: 10.1192/bjo.2022.528 (PMC9301776; doi:10.1192/bjo.2022.528)
Supplement: Supplementary file 1 [file S2056472422005282sup001.docx]

**Supplementary Materials**

**Semi-Structured Interview Topic Guide - Version 1.0 3December2018**

**ARENA EVENT RESEARCH: SOCIAL INFLUENCES ON RECOVERY ENQUIRY (SIRE)**

**Preamble**

- Interviewer Explains role/accountability
- Review Interviewee consent and written consent forms
- Safeguarding contingencies in the event of identifying any risk/confidentiality related issues
- Tape recording explained and consent recorded
- Provide rationale for interview, for example:

*’The Manchester bombing was very distressing for many who attended the Arena that night. We’re interested in how you’ve been coping since the event and what you have found helpful in recovering after the event. We’re particularly interested in hearing about the support you might have received from your social network for example. from your friends, family and other groups as well as things that might have added to your stress or delayed your recovery.’*

**Part** **I: SOCIAL CONTEXT BEFORE THE EVENT**

1. **How would you describe what life was like for you before the Arena event ?**

Prompt: living arrangements? close family relationships? employment/education status? social life and leisure activities?

**Part II: EXPERIENCE AT THE ARENA AND IMMEDIATELY AFTER**

**Preface:** remind no requirement to discuss any distressing events in detail

1. **Going in to as much detail as you feel comfortable with, what did you experience at the Arena that night?**

Prompt: Immediate thoughts, feelings and behaviour at the time?

**3. What impact did it have on you in the early days and weeks after the event?**

Prompt: Any immediate impacts on daily life and relationships?

**4. How did you cope at this time?**

Prompt: Expected/perceived/actual sources of support? and how they were helpful or unhelpful?

**Part III: SOCIAL INFLUENCES ON COPING/RECOVERY**

**Preface:** *’So far, we’ve talked about your experiences before and immediately the event. Now can we go on to look at if and how things might have changed over the months that followed? And talk about where you are now?’*

**5. Looking back, who or what has helped you cope or recover from the event?**

Prompt: Explore all potential sources of support:

- Close interpersonal relationships
- Family support
- Wider sources of support eg local community, collective events and social networks, services, therapy, and any other?

**Semi-Stuctured Interview Topic Guide - Version 1.0 3December2018**

- Emotional Support – i.e. any other behaviour or experiences fostering feelings of comfort, being cared for by others?
- Informational/cognitive support - ie any relevant info or advice to help coping, understand crisis, adjust to changes, normalisation messages, reassuring explanations of ordinary reactions, use of Self-management materials - either on line or other?
- Instrumental Aid/Material Support – i.e. any goods and services helped to solve practical problems?
- Personal Appraisal support - ie having strengths and assets acknowledged by others?

Prompt: Explore how each type of support has made a difference - when? why? how?

**6. Is there anyone or anything that has hindered you in your coping and recovery?**

Prompt: How it has affected it - when? why? how?

**7. Have you given support to anyone else affected by the event?**

Prompt: And did this help them and/or you?

**8. Have you had any other stressful experiences or events since the Arena event?**

Prompt: Explore any recovery related social problems? e.g. changes in close relationships, separation from family, social networks etc.

Prompt: impact of any other secondary stressors? e.g. financial hardships, health problems, access work/education, lack information/services etc

**9. How would you describe your overall wellbeing now?**

Prompt: emotional and physical wellbeing? current functioning? ongoing impact on close relationships? and ongoing impact on wider social contexts?

**10. Looking back - Anything else that could or should have be done that might have helped you?**

**11. Has the event and all that’s happened to you since changed the way you view yourself now? Or how you see your future?**

**Ending**

- Thank you for your time and help. Any further points you would like to make?
- Any questions?
- How do you feel now the interview has completed? (Prompt for distress)
- Signpost to appropriate support services if required
- Review consent and confidentiality issues
- Would you like to be forwarded research findings when analysis completed?
- Final thanks for assisting with the research
